# Response to letter regarding “low apolipoprotein B and LDL-cholesterol are associated with the risk of cardiovascular and all-cause mortality: a prospective cohort”

**DOI:** 10.1080/07853890.2025.2586149

**Published:** 2025-12-03

**Authors:** Xiaolin Yu, Yujuan Yuan, Xiangyu Dong, Zulipiyemu Xier, Ling Ma, Hui Peng, Yining Yang

**Affiliations:** ^a^ Department of Cardiology, People’s Hospital of Xinjiang Uyghur Autonomous Region, Urumqi, China; ^b^ Xinjiang Key Laboratory of Cardiovascular Homeostasis and Regeneration Research, Urumqi, China

We sincerely thank the authors of the Letter [1] for their careful reading of our article [2] and for raising valuable points that allow us to further clarify our methods and interpretation. Below, we address each concern in turn.

## 1. Missing covariate data and sample size reliability

Participants with missing LDL-C or apoB values were excluded, as shown in the study flowchart. For other covariates, we applied multiple imputation, a widely accepted method in epidemiology that minimizes bias and preserves sample size under the assumption of missing-at-random. We acknowledge that this was not clearly stated in the main text, and we are grateful for the opportunity to clarify it here.

## 2. Study population description

We agree with the point regarding terminology. As participants younger than 18 years were excluded, our study population should more precisely be described as ‘adults from the general population’. We thank the authors for this observation.

## 3. Definitions of covariates

Detailed definitions of covariates were based on NHANES coding and established literature, and have been provided in our supplementary material. To summarize:

Smoking status: Never smokers (<100 cigarettes lifetime), former smokers (≥100 cigarettes lifetime but not currently smoking), and current smokers (≥100 cigarettes lifetime and currently smoking).

Alcohol intake was classified into four categories: never (0 drinks per week), light (<1 drink per week), moderate (1–7 drinks per week), and heavy (≥8 drinks per week), a categorization previously applied in the Health, Aging, and Body Composition Study [3].

Comorbidities: Self-reported physician diagnoses from the NHANES Medical Conditions Questionnaire (MCQ), consistent with NHANES coding protocols and widely used in prior analyses.

## 4. Reported hazard ratios (HRs)

We acknowledge the need for precise wording when interpreting HRs: HR > 1 indicates higher risk, whereas HR < 1 indicates lower risk. In our analysis, apoB <90 mg/dL served as the reference. Thus, the HR of 0.79 (95% CI 0.69–0.89) for apoB ≥90 mg/dL indicates lower all-cause mortality in this group, which conversely means that low apoB (<90 mg/dL) was associated with a higher mortality hazard. We recognize that the phrasing in the text may have caused confusion, and we appreciate the opportunity to clarify.

Similarly, the HR for cerebrovascular death (2.13, 95% CI 1.01–4.49) indeed represents an increased risk and should be interpreted as such.

In the combined analysis of LDL-C and apoB, the correct HR for the LDL-C 70–99 mg/dL group is 1.37, as shown in Table 2. These clarifications do not affect the study’s overall conclusions.

## 5. Potential confounding factors (exercise and diet)

We agree that lifestyle factors such as physical activity and dietary intake are important determinants of lipid levels. However, due to the complexity and missingness of these variables in NHANES, we did not adjust for them in our primary models. We acknowledged this as a study limitation and emphasized that future research should incorporate these variables where feasible.

## 6. Subgroup analyses

We appreciate the suggestion to examine subgroups by demographic or lifestyle factors (e.g. by gender, age, ethnicity, BMI, education level, alcohol consumption, smoking status, etc.). Our current study’s primary aim was overall associations, and exploratory subgroup analyses would require careful control for multiple comparisons and sufficient sample size in each stratum. We plan to investigate interactions and stratified associations in future work when more data accumulate, to ensure adequate statistical power for those analyses.

## Conclusion

We thank the authors of the Letter once again for their thoughtful comments. Their input provides an opportunity to clarify our methodology and interpretation, thereby strengthening the understanding and applicability of our findings.

## Data Availability

Data sharing is not applicable to this article as no new data were created or analyzed in this study.

## References

[1] Yu X, Yuan Y, Dong X, et al. Low apolipoprotein B and LDL-cholesterol are associated with the risk of cardiovascular and all-cause mortality: a prospective cohort. Ann Med. 2025;57(1):2529565. doi: 10.1080/07853890.2025.2529565. 40642933 PMC12258213

[2] Yang W. Regarding: “Low apolipoprotein B and LDL-cholesterol are associated with the risk of cardiovascular and all-cause mortality: a prospective cohort.” Ann Med. 2025;57(1):2566876. doi: 10.1080/07853890.2025.2566876. 41021274 PMC12481532

[3] Maraldi C, Volpato S, Kritchevsky SB, et al. Impact of inflammation on the relationship among alcohol consumption, mortality, and cardiac events: the health, aging, and body composition study. Arch Intern Med. 2006;166(14):1490–1497. doi: 10.1001/archinte.166.14.1490. 16864759

